# Chemoselective Polymerization of Polar Divinyl Monomers with Rare-Earth/Phosphine Lewis Pairs

**DOI:** 10.3390/molecules23020360

**Published:** 2018-02-08

**Authors:** Pengfei Xu, Lei Wu, Liqiu Dong, Xin Xu

**Affiliations:** 1 Key Laboratory of Organic Synthesis of Jiangsu Province, College of Chemistry, Chemical Engineering and Materials Science, Soochow University, Suzhou 215123, China; 20164009015@stu.suda.edu.cn (P.X.); 1509401010@stu.suda.edu.cn (L.W.); dongliqiufrank@gmail.com (L.D.); 2 Suzhou International Academy, BFSU, Suzhou 215200, China

**Keywords:** Lewis pairs, rare-earth, phosphines, chemoselective polymerization

## Abstract

This work reports the chemoselective polymerization of polar divinyl monomers, including allyl methacrylate (AMA), vinyl methacrylate (VMA), and 4-vinylbenzyl methacrylate (VBMA), by using simple Lewis pairs comprised of homoleptic rare-earth (RE) aryloxide complexes RE(OAr)_3_ (RE = Sc (**1**), Y (**2**), Sm (**3**), La (**4**), Ar = 2,6-*t*Bu_2_C_6_H_3_) and phosphines PR_3_ (R = Ph, Cy, Et, Me). Catalytic activities of polymerizations relied heavily upon the cooperation of Lewis acid and Lewis base components. The produced polymers were soluble in common organic solvents and often had a narrow molecular weight distribution. A highly syndiotactic poly(allyl methacrylate) (PAMA) with *rr* ~88% could be obtained by the scandium complex **1**/PEt_3_ pair at −30 °C. In the case of poly(4-vinylbenzyl methacrylate) (PVBMA), it could be post-functionalized with PhCH_2_SH. Mechanistic study, including the isolation of the zwitterionic active species and the end-group analysis, revealed that the frustrated Lewis pair (FLP)-type addition was the initiating step in the polymerization.

## 1. Introduction

The post-modification of polymers containing reactive vinyl groups offers a great opportunity to obtain many advanced functional materials [1,2,3,4,5,6,7]. The chemoselective polymerization of polar divinyl monomers represents one of the most powerful methods to produce such polymers. Among the enormous efforts which have been made so far, anionic [8,9] and radical [10,11,12,13] polymerization have been extensively investigated. However, achieving complete chemoselectivity and preventing crosslinking during the whole process remains a great challenge, especially in the later stage of the polymerization [14]. To maintain the chemoselectivity of the polymerization at the later stage, harsh reaction conditions (e.g., low temperature or suitable Lewis acid additive) were often required [15,16]. Limited catalyst systems have been developed for the chemoselective polymerization of polar divinyl monomers under mild conditions [15]. Chen’s group recently reported an elegant example of the chemoselective polymerization of polar divinyl monomers by using chiral ansa-zirconocene enolates, affording a highly stereotactic polymer [17,18]. The same group achieved complete chemoselectivity in the polymerization of multivinyl-functionalized *γ*-butyrolactones by utilizing *N*-heterocyclic carbene (NHC) catalysts [19]. Chemoselective polymerization of allyl methacrylate was also realized by a yttrium monoalkyl complex [20]. 

**Scheme 1:**
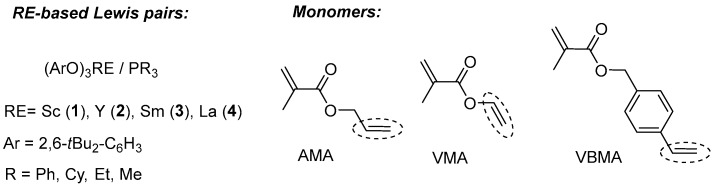
Rare-earth (RE)-based Lewis pairs and monomers examined in this work. AMA: allyl methacrylate; VBMA: 4-vinylbenzyl methacrylate; VMA: vinyl methacrylate.

Inspired by flourishing frustrated Lewis pairs (FLPs) chemistry [21,22], Lewis pair polymerization (LPP) of polar alkenes by main-group Al- or B-based Lewis pairs was successfully achieved in recent years [23,24,25,26,27,28]. We reported the polymerization of methyl methacrylate (MMA) and its cyclic analogues by using the intramolecular cationic rare-earth (RE)-based Lewis pairs [29]. Subsequently, enhanced polymerization activity and extended monomer scope were realized by utilizing simple intermolecular Lewis pairs which are composed of homoleptic rare-earth metal tris-aryloxides RE(OAr)_3_ and phosphines PR_3_ [30]. We herein found that such rare-earth/phosphine systems also enabled the chemoselective polymerization of polar divinyl monomers, affording polymers with the retention of pendant C=C bonds (Scheme 1). These results will be described and discussed in this article.

## 2. Results and Discussion

First, we examined the polymerization of allyl methacrylate (AMA) using our RE-based Lewis pairs. Polymerization experiment was conducted with the **1**/PPh_3_ pair in a Lewis acid/Lewis base (LA/LB) ratio of 2 and a [AMA]/[LB] ratio of 200, and no polymer was yielded up to 24 h (Table 1, entry 1). Replacement of the Lewis base PPh_3_ with a more basic tris-alkyl phosphine tricyclohexylphosphine (PCy_3_) led to a 100% monomer conversion after 25 min (Table 1, entry 2). The ^1^H-NMR spectrum of the resulting polymer clearly showed that the allylic C=C bond remained unreacted. The high M_n_ (12.3 × 10^4^ g/mol) of the polymer probably resulted from the steric hindrance of the zwitterionic propagating species [26,30]. We thus performed the same polymerization with the less-sterically hindered triethylphosphine (PEt_3_), which yielded a polymer with a more controlled M_n_ of 5.03 × 10^4^ g/mol and a syndiotacticity of 79.9% (Table 1, entry 3). A higher syndiotactic PAMA (*rr* value of 87.8%) could be obtained by conducting the polymerization at −30 °C, albeit with a low activity (Table 1, entry 4). Even switching to a less-sterically hindered trimethylphosphine (PMe_3_) did not obviously change the polymerization results (Table 1, entry 5). It is well-established that the catalytic reactivity of rare-earth metal complexes highly relies on the metal ionic radii [31,32]. Subsequently, the larger metals in the series were employed as the Lewis acid components in the polymerization, with PEt_3_ as the Lewis base component. Excitingly, all of the polymerizations showed complete chemoselective behaviors. Quantitative monomer consumptions were achieved in 3 min for **2** (Table 1, entry 6) and 1 min for **3** and **4** (Table 1, entries 7 and 8), respectively. The polydispersity (PDI) of the produced polymer was also narrow (1.34–1.42). This showed that the polymerization activity increased with the increasing of metal ionic radius (La > Sm > Y > Sc). For the **4**/PEt_3_ pair, with a very small amount of catalyst loading (0.25 mol %), quantitative monomer conversion could be achieved in 5 min, producing syndiotactic-rich PAMA with high molecular weight (Table 1, entry 9). Notably, control experiments by using either the Sc complex **1** or the La complex **4** alone in the AMA polymerization under standard conditions led to no monomer conversion, even up to 24 h (Table S1, entries 12 and 13).

We next examined our RE/P catalytic system for the polymerization of VMA, an analogue of AMA. The **1**/PEt_3_ pair exhibited good activity for the polymerization of VMA, achieving 100% monomer conversion after 10 min. The produced PVMA had a M_n_ of 5.62 × 10^4^ g/mol and a PDI of 1.34 (Table 1, entry 10). Switching to trimethylphosphine (PMe_3_) did not noticeably change the polymerization results (Table 1, entry 11). The La complex **4**/PEt_3_ pair also showed high activity in the polymerization, which consumed 100 equivalent monomers within only 1 minute and yielded polymer with a relatively broad PDI of 1.76 (Table 1, entry 12). We then conducted the same polymerization in CH_2_Cl_2_, and the resulting polymer showed a narrower PDI of 1.43 (Table S1, entry 17).

A more challenging monomer was VBMA, because the relative reactivity ratio for the methacrylic C=C and the styrenic vinyl group is quite small (*r*_1_/*r*_2_ = 1.2) [33,34]. Gratifyingly, our intermolecular RE/P systems were found to be active for the chemoselective polymerization of VBMA with the retention of styrenic C=C bonds. This monomer was quantitatively converted to PVBMA in 45 min by the Sc complex **1**/PEt_3_(or PMe_3_) pair (Table 1, entries 13 and 14). When using the La complex **4**/PEt_3_ pair as a catalyst, polymerization proceeded rapidly and gave 100% monomer conversion in only 5 min (Table 1, entry 15). The resulting PVBMA had a syndiotacticity of 72.1% *rr*.

**Scheme 2:**
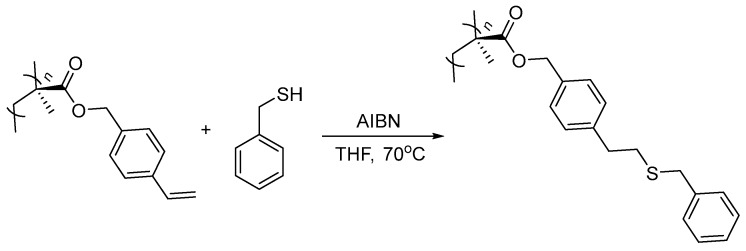
Post-functionalization of PVBMA with PhCH_2_SH. AIBN: 2,2′-azobis(2-methylpropionitrile).

Since the polymer obtained in the current polymerization still possessed reactive pendant C=C bonds, we decided to examine if it could undergo post-functionalization reaction. Treatment of the PVBMA with a stoichiometric excess amount of benzyl thiol (PhCH_2_SH), using catalytic amount of 2,2′-azobis(2-methylpropionitrile) (AIBN) as the initiator, afforded functionalized product as expected (Scheme 2). The produced polymer had an increased M_n_ of 6.80 × 10^4^ g/mol and a broader PDI of 3.27, which indicated some degree of cross-linking during the post-functionalization reaction. The ^1^H-NMR spectrum of the product showed a full conversion of the pendant C=C double bonds, which was evidenced by complete disappearance of the signals corresponding to the olefinic protons in PVBMA (δ 6.64, 5.71 and 5.22 ppm) and appearance of new saturated CH_2_ signals at δ 2.76 and 2.61 ppm ascribed to [S]CH_2_CH_2_[Ph] units (Figure S10).

**Scheme 3:**
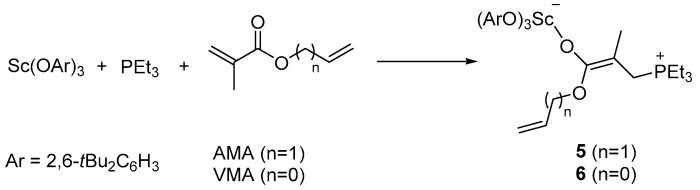
1,4-Addition reactions of the Sc/P Lewis pair to AMA and VMA.

**Figure 1:**
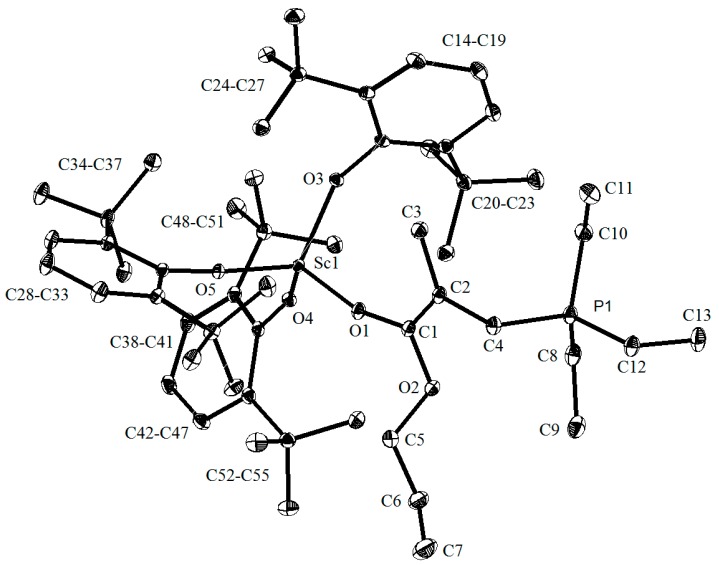
Molecular structure of the AMA addition product **5**. Hydrogen atoms are omitted for clarity, ellipsoids are drawn at 30% probability. Selected bond lengths (Å) and angles(^o^): Sc1-O1 1.9671(11); O1-C1 1.3117(19); C1-C2 1.350(2); C2-C3 1.498(2); C2-C4 1.516(2); C5-C6 1.482(3); C6-C7 1.309(3); P1-C4 1.8091(17); O1-C1-O2 115.40(14); O1-C1-C2 127.30(16); O2-C1-C2 117.29(14); C1-C2-C3 122.71(15); C4-C2-C3 117.94(15); C4-C2-C1 119.25(15).

To explore the mechanism of the current polymerizations, we investigated stoichiometric reactions of the Sc complex **1**/PEt_3_ pair with the abovementioned polar divinyl compounds. First, treatment of the **1**/PEt_3_ pair with AMA in a 1:1 molar ratio in toluene at room temperature yielded the addition product **5** as a white crystalline solid (Scheme 3, 79% yield). Complex **5** could be readily characterized by NMR for the phosphonium cation Et_3_P^+^ [δ 36.9 ppm in ^31^P-NMR; δ 2.05 (m, CH_2_), 1.25 (m, CH_3_) ppm in ^1^H-NMR] and for the ester enolate moiety [δ 5.84 (m, 1H, CH=CH_2_), 5.08 (m, 2H, CH=CH_2_), 4.32 (m, 2H, OCH_2_) ppm in ^1^H-NMR]. The molecular structure of **5** was also confirmed by single-crystal X-ray diffraction analysis (Figure 1), which clearly showed that a kinetically-controlled 1,4-addition reaction occurred during the reaction to produce *trans*-configured product with an enolate moiety (Sc1-O1 1.9671(11) Å, O1-C1 1.3117(19) Å, C1-C2 1.350(2) Å, O1-C1-C2-C4 4.284(153)°) and an unreacted terminal C=C bond (C6-C7 1.309(3) Å). The analogous reaction of the **1**/PEt_3_ pair with VMA also yielded the *trans* Sc/P 1,4-addition complex **6** as a white crystalline solid (77% yield, Scheme 3), which was also comprehensively characterized by multinuclear NMR spectroscopy, elementary analysis, and single-crystal X-ray diffraction (Figure S7). Complex **6** showed similar structural features and spectroscopy properties to those of the AMA addition product **5** (for details, see the Supporting Information). Notably, the 1,4-addition reaction occurred through the conjugated C=C bond, leaving the non-conjugated C=C intact, which is at the origin of the high level of chemoselectivity observed in the polymerization reaction. Subsequently, the polymerizations with the isolated complexes **5** and **6** as initiators were performed. Complex **5** exhibited a low activity for the polymerization of AMA in CH_2_Cl_2_, achieving 32% monomer conversion after 24 h (Table 2, entry 1). On the other hand, highly active polymerization could be achieved by adding an additional equivalent of the Lewis acidic Sc complex **1**, affording a 100% monomer conversion in 20 min (Table 2, entry 2). The polymerization of VMA catalyzed by complex **6** with [VMA]/[6] = 100 in CH_2_Cl_2_ led to no monomer conversion up to 24 h (Table 2, entry 3). Nevertheless, a quantitative monomer consumption was realized in 20 min by adding an additional equivalent of the Sc complex **1** (Table 2, entry 4). These observations indicated that the polymerization requires another equivalent of Lewis acid to activate monomer, which is consistent with the activated monomer propagation mechanism [23,24,25,26,29,30].

Finally, we also conducted an oligomerization reaction using the scandium complex **1**/PEt_3_ pair in an [AMA]/[PEt_3_] molar ratio of 20. After quenching with wet MeOH, the resulting oligomer was then analyzed by MALDI-TOF MS spectrum (Figure S11). It clearly showed a major series of mass ions with a repeat unit of 126.10, which corresponded to the mass of the AMA monomer. A plot of *m*/*z* values of this series versus the number of AMA repeat units produced a straight line with a slope of 126.10 and an intercept of 119.47 (Figure S12). The intercept is equal to the sum of H^+^ and PEt_3_, suggesting that the produced oligomer has a structural formula of Et_3_P^+^-(AMA)_n_-H, where the phosphorus atom is attached to AMA. Thus, the polymerization was initiated by an FLP-type 1,4-addition reaction of the RE/P Lewis pair to the monomer.

## 3. Materials and Methods 

### 3.1. General Information

All manipulations were performed under a dry Argon atmosphere using standard Schlenk techniques or in a nitrogen-filled glovebox. Solvents (including deuterated solvents used for NMR) were dried and distilled prior to use. NMR spectra were recorded on a Bruker 400 MHz spectrometer (Bruker (Beijing) Scientific Technology Co., Ltd., Beijing, China). Chemical shifts were reported as δ units with reference to the residual solvent resonance or an external standard. The assignments of NMR data were supported by 1D and 2D-NMR experiments. Elemental analysis data was recorded on a Carlo-Erba EA-1110 instrument (CE Instruments Ltd, Wigan, UK). AMA and VMA were purchased from TCI (Tokyo Chemical Industry Co., Ltd., Tokyo, Japan). The VBMA was synthesized by following the literature procedures [35]. These monomers were dried over CaH_2_ and distilled prior to use. Phosphines—including PPh_3_, PCy_3_, PEt_3_, and PMe_3_—were purchased from Alfa Aesar (Alfa Aesar (China) Chemical Co., Ltd., Shanghai, China) and used as received. RE(OAr)_3_ (RE = Sc (**1**), Y (**2**), Sm (**3**), La (**4**), Ar = 2,6-*t*Bu_2_C_6_H_3_) were synthesized by following the literature procedures [36]. The AIBN was purchased from TCI and purified by recrystallization from methanol prior to use.

### 3.2. Procedures and Compound Characterization

#### 3.2.1. Preparation and Characterization of Complex **5**

To a solution of Sc(OAr)_3_ (132 mg, 0.2 mmol) and AMA (25 mg, 0.2 mmol) in toluene (0.5 mL), PEt_3_ (24 mg, 0.2 mmol, in 0.5 mL of toluene) was added. After standing at room temperature for 1 h, a large amount of colorless crystalline solid was precipitated, which was then washed with hexane (2 × 0.5 mL) to give complex **5** (150 mg, 79%). Crystals suitable for the single-crystal X-ray structure analysis were grown from a benzene solution of **5** at room temperature. ^1^H-NMR (400 MHz, CD_2_Cl_2_, 298 K): δ = 7.04 (m, 6H, *m*-O*Ar*), 6.50 (m, 3H, *p*-O*Ar*), 5.84 (m, 1H, CH=CH_2_), 5.08 (m, 2H, CH=CH_2_), 4.32 (m, 2H, OCH_2_), 2.88 (d, ^2^*J*_PH_ = 11.1 Hz, 2H, PC*H*_2_C=), 2.05 (m, 6H, PC*H*_2_CH_3_), 1.52 (m, 3H, C*H*_3_C=), 1.42 (s, 54H, C(CH_3_)_3_), 1.25 (m, 9H, PCH_2_C*H*_3_). ^13^C{^1^H} NMR (101 MHz, CD_2_Cl_2_, 298 K): δ = 163.4 (*i*-O*Ar*), 159.5 (d, ^3^*J*_PC_ = 8.9 Hz, O*C*=), 139.0 (*o*-O*Ar*), 135.6 (CH=CH_2_), 125.1 (*m*-O*Ar*), 116.8 (CH=CH_2_), 115.8 (*p*-O*Ar*), 68.3 (d, ^5^*J*_PC_ = 1.4 Hz, O*C*H_2_), 67.2 (d, ^2^*J*_PC_ = 9.6 Hz, CH_3_C=), 35.8 (C(CH_3_)_3_), 32.7 (C(CH_3_)_3_), 24.1 (d, ^1^*J*_PC_ = 45.7 Hz, P*C*H_2_C=), 18.5 (d, ^3^*J*_PC_ = 1.0 Hz, CH_3_C=), 12.5 (d, ^1^*J*_PC_ = 47.1 Hz, PCH_2_CH_3_), 6.0 (d, ^2^*J*_PC_ = 5.1 Hz, PCH_2_CH_3_). ^31^P{^1^H} NMR (162 MHz, CD_2_Cl_2_, 298 K): δ = 36.9 (ν_1/2_ ~ 5 Hz). Elemental Analysis: calculated for C_55_H_88_O_5_PSc·0.5C_7_H_8_: C, 73.86; H, 9.75. Found: C, 73.75; H, 9.30.

X-ray crystal structure analysis of complex **5**: formula C_55_H_88_O_5_PSc·C_6_H_6_, M = 944.24, colorless, 0.25 × 0.25 × 0.20 mm, a = 18.7281(8), b = 18.5836(7), c = 16.1009(7) Å, *β* = 100.6070°, *V* = 5507.9(4) Å^3^, ρ_calc_ = 1.139 g cm^−3^, μ = 0.207 mm^−1^, Z = 4, monoclinic, space group P2(1)/c, λ = 0.71073 Å, T = 120(2) K, Multi-scan, 87,725 reflections collected (±h, ±k, ±l), 12,616 independent (R(int) = 0.0565) and 9925 observed reflections [I > 2σ(I)], 608 refined parameters, R = 0.0381, wR2 = 0.1267, max. (min.) residual electron density 0.550 (−0.625) e.Å^−3^, hydrogen atoms were calculated and refined as riding atoms.

#### 3.2.2. Preparation and Characterization of Complex **6**

Following the procedure described for **5**, reaction of Sc(OAr)_3_ (132 mg, 0.2 mmol) and VMA (22 mg, 0.2 mmol) with PEt_3_ (24 mg, 0.2 mmol) gave **6** as colorless crystals (151 mg, 77%). Crystals suitable for the X-ray single-crystal structure analysis were grown from a benzene solution of **6** at room temperature. ^1^H-NMR (400 MHz, CD_2_Cl_2_, 298 K): δ = 7.05 (m, 6H, *m*-O*Ar*), 6.98 (overlapped, 1H, CH=CH_2_), 6.51 (m, 3H, *p*-O*Ar*), 4.36 (m, 1H, CH=CH_2_), 3.99 (m, 1H, CH=CH_2_), 2.90 (d, ^2^*J*_PH_ = 11.5 Hz, 2H, PCH_2_C=), 2.07 (m, 6H, PCH_2_CH_3_), 1.48 (d, ^4^*J*_PH_ = 2.9 Hz, 3H, CH_3_C=), 1.41 (s, 54H, C(CH_3_)_3_), 1.28 (m, 9H, PCH_2_CH_3_). ^13^C{^1^H} NMR (101 MHz, CD_2_Cl_2_, 298 K): δ = 163.3 (*i*-O*Ar*), 157.3 (d, ^3^*J*_PC_ = 9.0 Hz, O*C*=), 148.1 (OCH=CH_2_), 139.0 (*o*-O*Ar*), 125.1 (*m*-O*Ar*), 116.0 (*p*-O*Ar*), 89.8 (OCH=CH_2_), 68.4 (d, ^2^*J*_PC_ = 9.4 Hz, CH_3_C=), 35.7 (*C*(CH_3_)_3_), 32.6 (C(*C*H_3_)_3_), 23.4 (d, ^1^*J*_PC_ = 45.7 Hz, P*C*H_2_C=), 18.2 (d, ^3^*J*_PC_ = 1.0 Hz, CH_3_C=), 12.4 (d, ^1^*J*_PC_ = 47.3 Hz, P*C*H_2_CH_3_), 6.0 (d, ^2^*J*_PC_ = 5.2 Hz, PCH_2_*C*H_3_). ^31^P{^1^H} NMR (162 MHz, CD_2_Cl_2_, 298 K): δ = 36.9 (ν_1/2_ ~ 5 Hz). Elemental Analysis: calculated for C_54_H_86_O_5_PSc·C_7_H_8_: C, 74.51; H, 9.64. Found: C, 74.66; H, 9.26.

X-ray crystal structure analysis of complex **6**: formula C_54_H_86_O_5_PSc·0.5C_6_H_6_, M = 930.21, colorless, 0.25 × 0.20 × 0.15 mm, a = 18.8243(9), b = 18.4532(8), c = 16.0590(8) Å, *β* = 100.998(2)°, *V* = 5475.9(4) Å^3^, ρ_calc_ = 1.128 gcm^−3^, μ = 0.208 mm^−1^, Z = 4, monoclinic, space group P2(1)/c, λ = 0.71073 Å, T = 120(2) K, Multi-scan, 118,961 reflections collected (±h, ±k, ±l), 12,526 independent (R(int) = 0.0499) and 10,196 observed reflections [I > 2σ(I)], 599 refined parameters, R = 0.0358, wR2 = 0.1288, max. (min.) residual electron density 0.601 (−0.713) e.Å^−3^, hydrogen atoms were calculated and refined as riding atoms.

#### 3.2.3. General Polymerization Procedures 

Polymerizations were performed in 20 mL oven-dried glass reactors at room temperature inside the glovebox. A predetermined amount of RE(OAr)_3_ (2 equiv.) was first dissolved in the solvent and monomer. Then, the polymerization was started by rapid addition of a solution of Lewis base via a pipette to the above solution under vigorous stirring. After the measured time interval, a 0.1 mL aliquot was taken from the reaction mixture and quickly quenched into a 4-mL vial containing 0.6 mL of undried “wet” CDCl_3_. The quenched aliquots were later analyzed by ^1^H-NMR to obtain the monomer conversion data. After the removal of the aliquot, the polymerization was immediately quenched by the addition of 5 mL 5% HCl-acidified methanol. The quenched mixture was poured into 50 mL methanol, stirred for 1 h, filtered, washed, and dried in a vacuum oven at 50 °C overnight to a constant weight to verify the polymer conversions determined by ^1^H-NMR.

#### 3.2.4. Polymer Characterizations

Polymer number (M_n_) and weight (M_w_) average molecular weights and polydispersity index (PDI = M_w_/M_n_) were measured by gel permeation chromatography (GPC) analyses carried out at 40 °C and a flow rate of 0.8 mL/min with DMF as the eluent, on a Waters University 1515 GPC instrument coupled with a Waters RI detector and equipped with four PLgel 5 μm mixed-C columns. The instrument was calibrated with 10 PMMA standards, and chromatograms were processed with Waters Empower 2 software. 

## 4. Conclusions

In summary, we have reported the complete chemoselective polymerization of a series of polar divinyl monomers under mild condition by the utilization of homoleptic rare-earth aryloxide-based Lewis pairs. Catalytic activities of polymerizations were highly dependent on the ionic radii of RE ions and electronic/steric profiles of the Lewis bases. The resulting polymers, bearing pendant C=C double bonds, could easily undergo post-functionalization with the thiol reagent. Remarkably, a highly syndiotactic PAMA with *rr* ~88% could be produced by such an RE-based Lewis pair system. The isolation of zwitterionic propagating species and the end-group analysis suggested that current polymerization was initiated by an RE/P FLP-type 1,4-addition, rather than RE covalent bond insertion or single-electron transfer in the traditional RE-catalyzed polymerizations.

## Figures and Tables

**Table 1:** Chemoselective polymerization of polar divinyl monomers with homoleptic rare-earth aryloxide-based Lewis pairs ^a^.

Entry	Monomer	LA	LB	[M]/[LB]	T (min)	Conv. (%)	M_n_ (10^4^ g/mol)	PDI (M_w_/M_n_)	M_n(theo)_^b^ (10^4^ g/mol)	*rr* (%)	*mr* (%)	*mm* (%)
1	AMA	1	PPh_3_	200	1440	0	-	-	-	-	-	-
2	AMA	1	PCy_3_	200	25	100	12.3	1.33	2.55	79.8	19.6	0.6
3	AMA	1	PEt_3_	200	15	100	5.03	1.23	2.54	79.9	19.5	0.6
4 ^c^	AMA	1	PEt_3_	200	1440	75	10.8	1.38	2.54	87.8	11.4	0.8
5	AMA	1	PMe_3_	200	30	90	5.09	1.23	2.53	79.2	20.0	0.8
6	AMA	2	PEt_3_	200	3	100	8.57	1.41	2.54	75.3	23.5	1.2
7	AMA	3	PEt_3_	200	1	100	9.16	1.34	2.54	75.0	23.7	1.7
8	AMA	4	PEt_3_	200	<1	100	10.0	1.42	2.54	72.5	26.0	1.5
9	AMA	4	PEt_3_	400	5	100	15.3	1.76	5.06	72.8	25.4	1.8
10	VMA	1	PEt_3_	100	10	100	5.62	1.36	1.13	75.6	22.6	1.8
11	VMA	1	PMe_3_	100	10	100	7.59	1.31	1.13	76.8	21.6	1.6
12	VMA	4	PEt_3_	100	1	100	4.74	1.76	1.13	69.3	29.1	1.6
13	VBMA	1	PEt_3_	100	45	100	2.55	1.87	2.03	75.0	22.5	2.5
14	VBMA	1	PMe_3_	100	45	100	2.42	1.99	2.03	76.2	22.8	1.0
15	VBMA	4	PEt_3_	100	5	100	2.92	2.00	2.03	72.1	26.3	1.6

^a^ Conditions: polymerizations were conducted at room temperature in toluene (V_monomer_/V_solvent_: 1:2) and a Lewis acid (LA)/Lewis base (LB) ratio of 2, where n_[LA]_ = 40 μmol. Monomer conversions were determined by ^1^H-NMR spectroscopy and confirmed by gravimetric methods; *rr*, *mr*, *mm* were measured by ^1^H-NMR spectroscopy. M_n_ and polydispersity (PDI) were determined by gel permeation chromatography (GPC) in *N*,*N*-dimethylformamide (DMF) relative to the poly(methyl methacrylate) (PMMA) standards; ^b^ M_n_(theo) = Mw(M) × [M]/[I] × conversion (%) + MW (chain-end groups); ^c^ Polymerization was conducted at −30 °C.

**Table 2:** Chemoselective polymerization of AMA and VMA with complexes **5** and **6**. ^a^

Entry	Monomer	Cat.	LA	[M]/[Cat.]	T (min)	Conv. (%)	M_n_ (10^4^ g/mol)	PDI (M_w_/M_n_)	*rr* (%)	*mr* (%)	*mm* (%)
1	AMA	5	-	100	1440	32	2.12	1.45	75.3	21.5	3.2
2	AMA	5	1	100	20	100	4.23	1.42	78.4	19.6	2.0
3	VMA	6	-	100	1440	0	-	-	-	-	-
4	VMA	6	1	100	20	100	4.89	1.42	77.4	21.7	0.9

^a^ Conditions: polymerizations were conducted at room temperature in CH_2_Cl_2_ (V_monomer_/V_solvent_: 1:2) by using 20 μmol catalyst. Monomer conversions were determined by ^1^H-NMR spectroscopy and confirmed by gravimetric methods; *rr*, *mr* and *mm* were measured by ^1^H-NMR spectroscopy. M_n_ and PDI were determined by GPC in DMF relative to the PMMA standards.

## Supplementary Materials

The Supplementary Materials are available online: supplementary materials contain part of experimental procedures, characterizations of the 1,4-addition complexes and oligomers; CCDC 1814864 (complex **5**) and 1814865 (complex **6**) contain the supplementary crystallographic data for this paper, these data can be obtained free of charge via www.ccdc.cam.ac.uk/data_request/cif, or by emailing data_request@ccdc.cam.ac.uk, or by contacting The Cambridge Crystallographic Data Centre, 12, Union Road, Cambridge CB2 1EZ, UK; Fax: +44-1223-336033.
